# A novel model of gestational diabetes: Acute high fat high sugar diet results in insulin resistance and beta cell dysfunction during pregnancy in mice

**DOI:** 10.1371/journal.pone.0279041

**Published:** 2022-12-15

**Authors:** Akansha Mishra, Simone Hernandez Ruano, Pradip K. Saha, Kathleen A. Pennington

**Affiliations:** 1 Department of Obstetrics and Gynecology and, Baylor College of Medicine, Houston, Texas, United States of America; 2 Department of Molecular and Cellular Biology, Baylor College of Medicine, Houston, Texas, United States of America; Nippon Medical School, JAPAN

## Abstract

Gestational diabetes mellitus (GDM) affects 7–18% of all pregnancies. Despite its high prevalence, there is no widely accepted animal model. To address this, we recently developed a mouse model of GDM. The goal of this work was to further characterize this animal model by assessing insulin resistance and beta cell function. Mice were randomly assigned to either control (CD) or high fat, high sugar (HFHS) diet and mated 1 week later. At day 0 (day of mating) mice were fasted and intraperitoneal insulin tolerance tests (ipITT) were performed. Mice were then euthanized and pancreata were collected for histological analysis. Euglycemic hyperinsulinemic clamp experiments were performed on day 13.5 of pregnancy to assess insulin resistance. Beta cell function was assessed by glucose stimulated insulin secretion (GSIS) assay performed on day 0, 13.5 and 17.5 of pregnancy. At day 0, insulin tolerance and beta cell numbers were not different. At day 13.5, glucose infusion and disposal rates were significantly decreased (p<0.05) in Pregnant (P) HFHS animals (p<0.05) suggesting development of insulin resistance in P HFHS dams. Placental and fetal glucose uptake was significantly increased (p<0.01) in P HFHS dams at day 13.5 of pregnancy and by day 17.5 of pregnancy fetal weights were increased (p<0.05) in P HFHS dams compared to P CD dams. Basal and secreted insulin levels were increased in HFHS fed females at day 0, however at day 13.5 and 17.5 GSIS was decreased (p<0.05) in P HFHS dams. In conclusion, this animal model results in insulin resistance and beta cell dysfunction by mid-pregnancy further validating its relevance in studying the pathophysiology GDM.

## Introduction

Gestational diabetes mellitus (GDM) is broadly defined as the diagnosis of diabetes during pregnancy [1]. GDM is one of the most common obstetrical complications affecting 7–18% of all pregnancies depending on demographics and diagnostic criteria [1–4]. Although GDM usually resolves after pregnancy, it is associated with lasting long-term health effects in mother and baby [2, 4]. While overweight and obese women are at an increased risk [5, 6] pregnant women with a normal BMI can develop GDM, and account for 28–32% of all GDM cases [5, 7, 8]. Studies are needed to discern causes of GDM, particularly in women without identifiable risk factors like obesity. This can be facilitated by animal models that recapitulate the key features of GDM.

Pregnancy is a natural state of insulin resistance [4, 9]. To compensate, beta cell mass expands, and insulin secretion increases in response to glucose, compared to a non-pregnant state [10–13]. Women with GDM have greater insulin resistance [14–16] coupled with an inadequate beta cell response that becomes apparent by mid-pregnancy [9, 17–20]. Previously we have shown that C57BL6/J mice exposed acutely to a high fat, high sugar (HFHS) diet, beginning 1 week before and during pregnancy have normal glucose tolerance prior to mating (d0), but exhibit glucose intolerance, decreased beta cell numbers, and decreased serum insulin levels at mid (d13.5) and late (d17.5) gestation [21]. HFHS dams also display dyslipidemia, including increased serum leptin levels and increased lipolysis [21, 22]. These symptoms resolve post-partum. In non-pregnant mice, the same dietary intervention does not cause glucose intolerance, and slightly increases insulin concentrations [21]. However, it is not known whether the acute HFHS diet exposure leads to insulin resistance in pregnant mice or impairs beta cell function, key characteristics of GDM.

Therefore, a detailed analysis of insulin resistance and sensitivity was performed in conscious, pregnant and non-pregnant control and HFHS-fed mice using the hyperinsulinemic euglycemic clamp method, the gold standard in assessing *in vivo* insulin resistance and sensitivity [23]. To our knowledge this is the first report using hyperinsulinemic euglycemic clamp in conscious pregnant mice, though one recent study used hyperinsulinemic euglycemic clamp in unconscious pregnant mice [24]. Insulin tolerance and pancreatic histology was assessed at day 0 of pregnancy (day of mating) to determine if insulin sensitivity and beta cell numbers were affected by HFHS feeding prior to mating. Finally, beta cell function was assessed by glucose stimulated insulin secretion assays at day 0, 13.5 and 17.5 of pregnancy. Together, these experiments demonstrate that acute HFHS diet feeding 1 week before and during pregnancy in mice results in GDM like symptoms and thus presents a valuable tool to further study the pathophysiology of GDM.

## Materials and methods

### Animals

All animal procedures were approved by the Baylor College of Medicine institutional animal care and use committee and performed in accordance with the NIH Guide for the Care and Use of Laboratory Animals.

**Fig 1:**
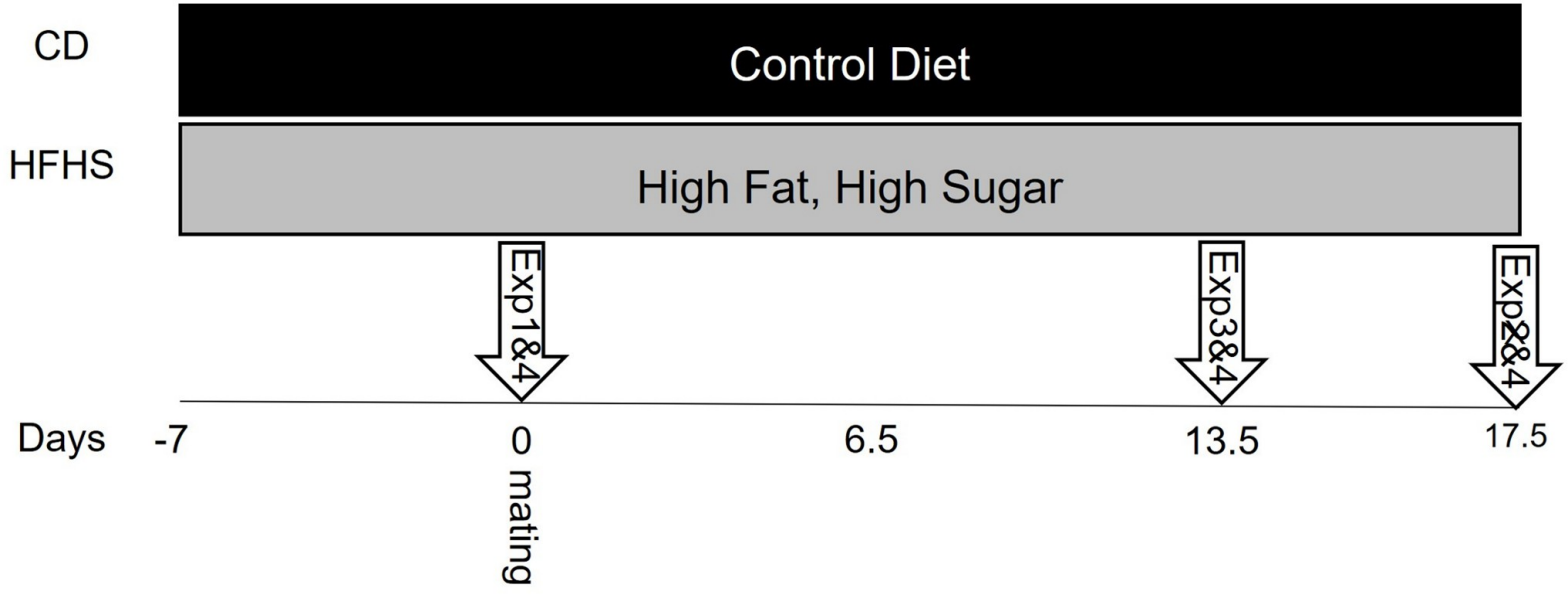
Overview of study design. Female mice were randomly assigned to CD of HFHS 7 days prior to mating. Experiments were performed at the time points as indicated by the arrows.

Seven week old C57BL/6J female mice were obtained from Jackson Laboratory (Bar Harbor, ME, USA). Mice were standardly housed at 18–23°C, 40–60% humidity, 14hour light/10hour dark cycle with ab libitum access to food and water and enrichment nestlets were provided in all cages. Mice were randomly placed on either a 10% kcal/fat, 0% kcal/sucrose control diet (CD, D12450K, Research Diets Inc., New Brunswick NJ) or a matched 45% kcal/fat, 17% kcal/sucrose HFHS diet (D12451, Research Diets Inc.) 1 week prior to and throughout pregnancy as previously described (See Fig 1 for study overview) [21]. Mice were mated to C57BL/6J proven breeder males for five days and the day of observed copulatory plug was identified as day 0.5 of pregnancy. Animal numbers for each experiment are by individual mouse and were based on our previously published work using this animal model [21, 22]. Experiment 1: At day 0 (day of mating) female mice were fasted and tested for intraperitoneal insulin tolerance (ipITT, CD Day 0: n = 6; HFHS, Day 0 n = 6). Mice were then euthanized and pancreata were collected for histological analysis (CD = 8, and HFHS = 8). Experiment 2: Hyperinsulinemic euglycemic clamps were performed in day 13.5 pregnant (P) conscious mice (P CD, n = 7; P HFHS, n = 7). Clamps were also performed on non-pregnant mice exposed to either CD (NP CD, n = 5) or HFHS (NP HFHS, n = 5) for 21 days (equivalent to day 13.5 of pregnancy diet exposure). Following the clamp procedure, mice were euthanized. Liver, fetus and placenta were collected, snap frozen in liquid nitrogen and stored at -80°C for measurement of glucose uptake. Experiment 3: Hyperinsulinemic euglycemic clamps were conducted in conscious mice at day 17.5 of pregnancy mice. As 5 of 6 mice began laboring on the day of the clamp procedure, the experiment was discontinued. Experiment 4: On Day 0, 13.5, and 17.5 pancreatic islets were isolated from both pregnant and non-pregnant females on either CD or HFHS diet and glucose stimulated insulin secretion assays were performed. Fetal and placenta weights were measured and recorded. For islet isolations, day 0 n = 4 CD and 4 HFHS mice, day 13.5 n = 6 NP CD, 9 P CD, 9 NP HFHS mice, and 9 P HFHS, day 17.5 n = 8 NP CD, 9 P CD, 6 NP HFHS, and 15 P HFHS mice.

### Intraperitoneal Insulin Tolerance Tests (ipITT)

We performed ipITT as previously described [25] and according to the National Mouse Metabolic Phenotyping Centers protocol for ipITT (https://mmpc.org/shared/document.aspx?id=84&doctype=Protocol). Briefly, mice were fasted for 4 hours, and then a baseline fasting blood glucose sample was obtained from a venous tail sample. An intraperitoneal injection of insulin (0.75 U/kg) was given. Blood glucose levels were obtained at 15, 30, 45, 60, 90 minutes post injection by removing the tail scab for blood collection. All blood glucose measurements were performed in duplicate using two ReliOn Prime Blood Glucose Monitoring System meters (Walmart, Bentonville, AR, USA).

### Pancreatic morphology

Immunohistochemistry was performed on pancreatic tissue from day 0 female mice to compare beta cell numbers and % proliferating cells as previously described [21, 26–28]. Briefly, pancreata were fixed in 4% paraformaldehyde, embedded in paraffin and 5 μm sections were cut at 100 μm intervals. Antigen retrieval was performed using 100 mM Tris-EDTA at 100°C for 30 minutes. Following antigen retrieval, tissue sections were blocked in 10% normal goat serum and then stained overnight at 4°C for insulin (ab7842, Abcam Inc, Cambridge, MA, USA) and Ki67 (BD Biosciences, San Jose, CA) at 1:1000 and 1:50 dilutions, respectively. Alexa Flour 488 goat anti-mouse IgG (ab150117, Abcam Inc) and Alexa Flour 594 goat anti-guinea pig IgG (A-11076, Invitrogen, Carlsbad, CA) were used for detection at 1:200 dilutions. Beta cells and Ki67 positive cells were manually counted in each pancreatic islet using ImageJ software (NIH) by an operator blinded to treatment group. All islets were counted in six independent tissue sections 100 μm apart from each other to estimate the average beta cell numbers and percent proliferating cells per islet for each mouse. Beta cell mass was also measured in each islet in six independent tissue sections 100 μm apart from each other using the ImageJ tracing feature as previously described [26].

### Hyperinsulinemic euglycemic clamps

Hyperinsulinemic euglycemic clamps were performed in conscious unrestrained non pregnant and day 13.5 pregnant mice using the insulin clamp technique in combination with HPLC purified [3-^3^H]glucose and [^14^C]2-deoxyglucose at the Baylor College of Medicine Mouse Metabolic and Phenotyping Core as previously described [29, 30]. For pregnant mice, surgery was performed at day 8.5 of pregnancy to insert catheters for clamp studies and mice were allowed to recover. At day 13.5 of pregnancy mice were fasted for 4 hours and hyperinsulinemic euglycemic clamps were performed. For non-pregnant mice, surgery was performed 5 days prior to the clamp procedure, which was performed 21 days post diet initiation. Both non-pregnant and pregnant mice received a priming dose (10 μCi) and then a constant infusion (0.1 μCi/min) of [3-^3^H] glucose for 3.5h. Blood samples were collected at 0, 50, and 60 min to measure the basal glucose production rate. After 1h infusion, mice were primed with regular insulin (bolus 20 mU/kg of body weight) followed by a 2h constant insulin infusion (5 mU/kg/min). Using a separate pump, 25% glucose was used to maintain the blood glucose level at 100–140 mg/dl, as determined every 10 min using a glucometer. Basal glucose production (BGP), peripheral glucose disposal rate (GDR), hepatic glucose production (HGP) and glucose infusion rate (GIR) were then calculated as previously described [31].

### Glucose uptake

To estimate insulin-stimulated glucose uptake in individual tissues, 2-[14C]-deoxyglucose (2DG) was administered as a bolus (10 uCi) 45 min before the end of the clamp procedure. Blood samples were collected 5, 10, 15, 25, 35 and 45 minutes after 2DG administration. At the end of the clamp procedure animals were euthanized and tissues were collected. Uptake of the non-metabolizable glucose analog 2DG in liver, whole fetus and placenta was measured as previously described [30]. Four fetuses and four placentas were pooled per dam for a single replicate. Glucose uptake in each tissue was calculated from the plasma 2-[^14^C] deoxyglucose profile fitted to a double exponential curve and tissue content of [^14^C] glucose--6 phosphate.

### Glucose Stimulated Insulin Secretion (GSIS) assays

Pancreatic islets were isolated from CD or HFHS fed non-pregnant (NP) or pregnant (P) female mice on day 0 (CD n = 4; HFHS n = 4) day 13.5 of pregnancy (NP CD n = 6, P CD n = 9, NP HFHS n = 9, P HFHS n = 9) and day 17.5 of pregnancy (NP CD n = 8, P CD n = 9, NP HFHS n = 6, P HFHS n = 12) as previously described [32] and used routinely in our labs. Briefly, pancreas was infused with collagenase, dissected from the animal and incubated in collagenase solution at 37°C. Once digestion was complete, samples were washed and ficoll gradient was applied to the isolated tissue. Cell pellets were resuspended in RPMI 1640. Islets were then picked, graded by size, and placed into culture overnight. The following day islets were selected by size with care taken to select islets of similar size for all groups and placed in tubes with low (1.8 mM) or high glucose (16.8 mM), 10 islets were used per tube and triplicate tubes were used per animal for low and high glucose concentrations. Media was collected following 30 min of culture to measure secreted insulin. Secreted and total insulin were measured using a Rat/Mouse Insulin ELISA (EMD Millipore, Billerica, MA, USA) according to manufacturer’s instructions as previously [21, 26, 33]. Percent secreted insulin was calculated and compared between groups to determine the effects of acute HFHS diet feeding on GSIS in islets from pregnant mice.

### Statistical analysis

Statistical analysis was performed using GraphPad Prism (La Jolla, CA, USA). A 2-way ANOVA with diet and time as factors was used to analyze ipITT data. Inverse area under the curve (AUC) was calculated for ipITT. Inverse AUC, weights, serum insulin, beta cell numbers, percent proliferating cells and beta cell mass were compared between control and HFHS-fed mice by Student’s t-test. Basal glucose, average glucose infusion rate, glucose disposal rate, and hepatic glucose production were analyzed by two-way ANOVA with diet and pregnancy status as factors, and Tukey test was used for post-hoc, pairwise comparisons. For day 0 GSIS a two-way ANOVA was performed with diet and insulin level as factors. For day 13.5 and 17.5 a three-way ANOVA was performed with diet, pregnancy status, and insulin level as factors. Fetal and placental glucose uptake and weights were compared between control and HFHS-fed mice by Student’s t-test.

## Results

### Acute high fat, high sugar diet does not alter insulin tolerance or beta cell numbers at day 0 of pregnancy

**Fig 2:**
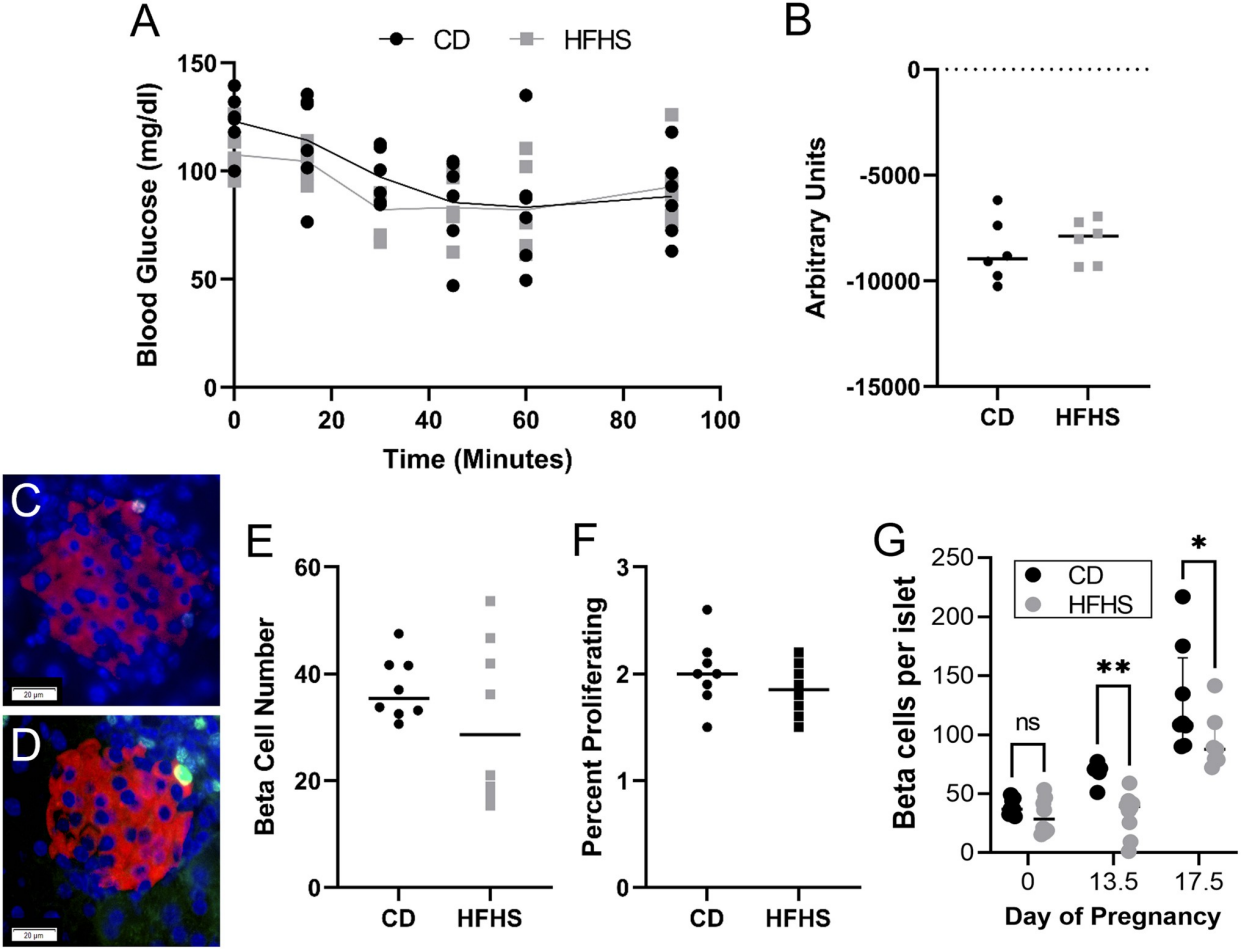
Insulin tolerance and beta cell numbers are normal at mating. Insulin tolerance curve (A) and inverse AUC (B) are not different between CD and HFHS fed mice on day 0 of pregnancy. Pancreatic islets were immuno-stained for insulin (red), Ki67 (green) and nuclei (dapi, blue). Representative images were chosen based on average islet cell numbers in (C) CD and (D) HFHS fed mice. Images were analyzed to determine mean number of (E) beta cells and (F) proliferating beta cells. For all ipITT measurements n = 6 CD and 6 HFHS, for day 0 histology n = 8 CD and 8 HFHS. (G) Combined beta cell number data from day 0 presented in this manuscript and day 13.5 and 17.5 data presented in our previous report [21]. Lines represent median values.

Previously, we reported that acute HFHS exposure did not result in glucose intolerance or altered serum insulin levels at day 0 of pregnancy [21]. Here, acute HFHS diet did not alter insulin tolerance compared to CD-fed female mice (Fig 2A and 2B). Histological examination of the pancreas showed no difference in beta cell numbers and percent proliferating beta cells. between CD and HFHS-fed mice at day 0 (Fig 2C–2F). Together with our previously published findings, this data indicates that at day 0 of pregnancy HFHS females have normal insulin resistance and beta cell function compared to CD fed females.

In our previous report we also showed that HFHS dams had decreased beta cell numbers by day 13.5, or mid pregnancy and that this decrease was still present at day 17.5 of pregnancy [21]. Here, we combined the beta cell data from all three time points to show the changes to beta cell numbers over time in our mouse model (Fig 2G). This figure clearly shows that CD dams display the expected increase in beta cell numbers during pregnancy, while this increase is impaired by mid pregnancy in HFHS dams.

### Acute high fat, high sugar diet results in insulin resistance during pregnancy

**Fig 3:**
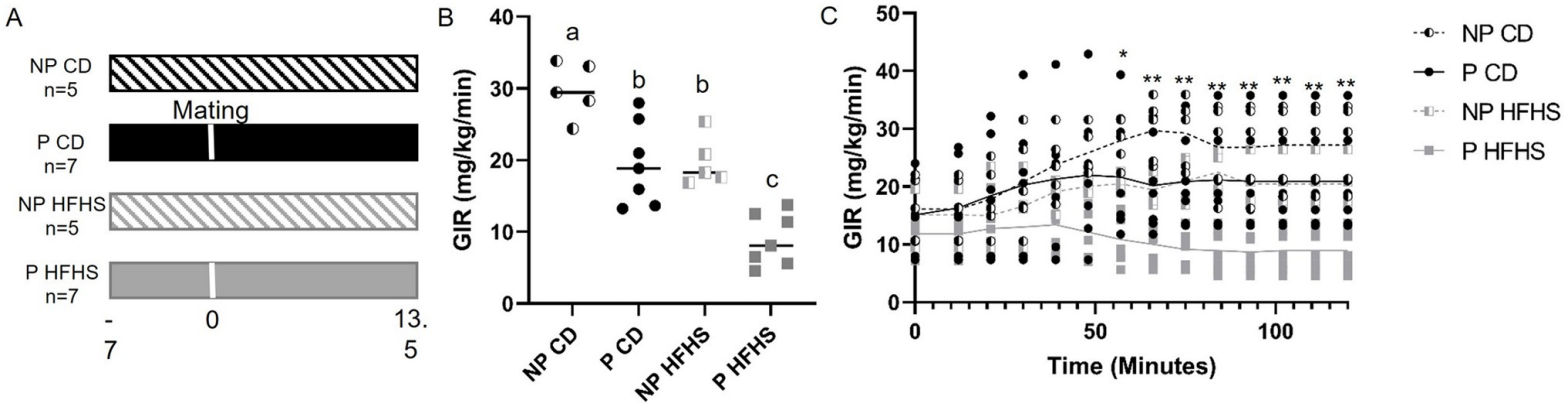
Dams exposed to acute HFHS feeding are insulin resistant. (A) Experimental timeline for hyperinsulinemic euglycemic clamp studies. (B) Average glucose infusion rate is significantly decreased in P HFHS dams compared to control dams as well as NP CD and NP HFHS females. (C) Glucose infusion rate over time is significantly reduced in P HFHS dams compared to all other groups, P CD and NP HFHS glucose infusion rates over time are significantly decreased compared to NP CD, but not different from each other. N = 5 NP CD, 7 P CD, 5 NP HFHS and 7 P HFHS mice. Difference subscripts represent differences among groups (p<0.01), *p<0.01; **p<0.01; Lines represent median values.

**Fig 4:**
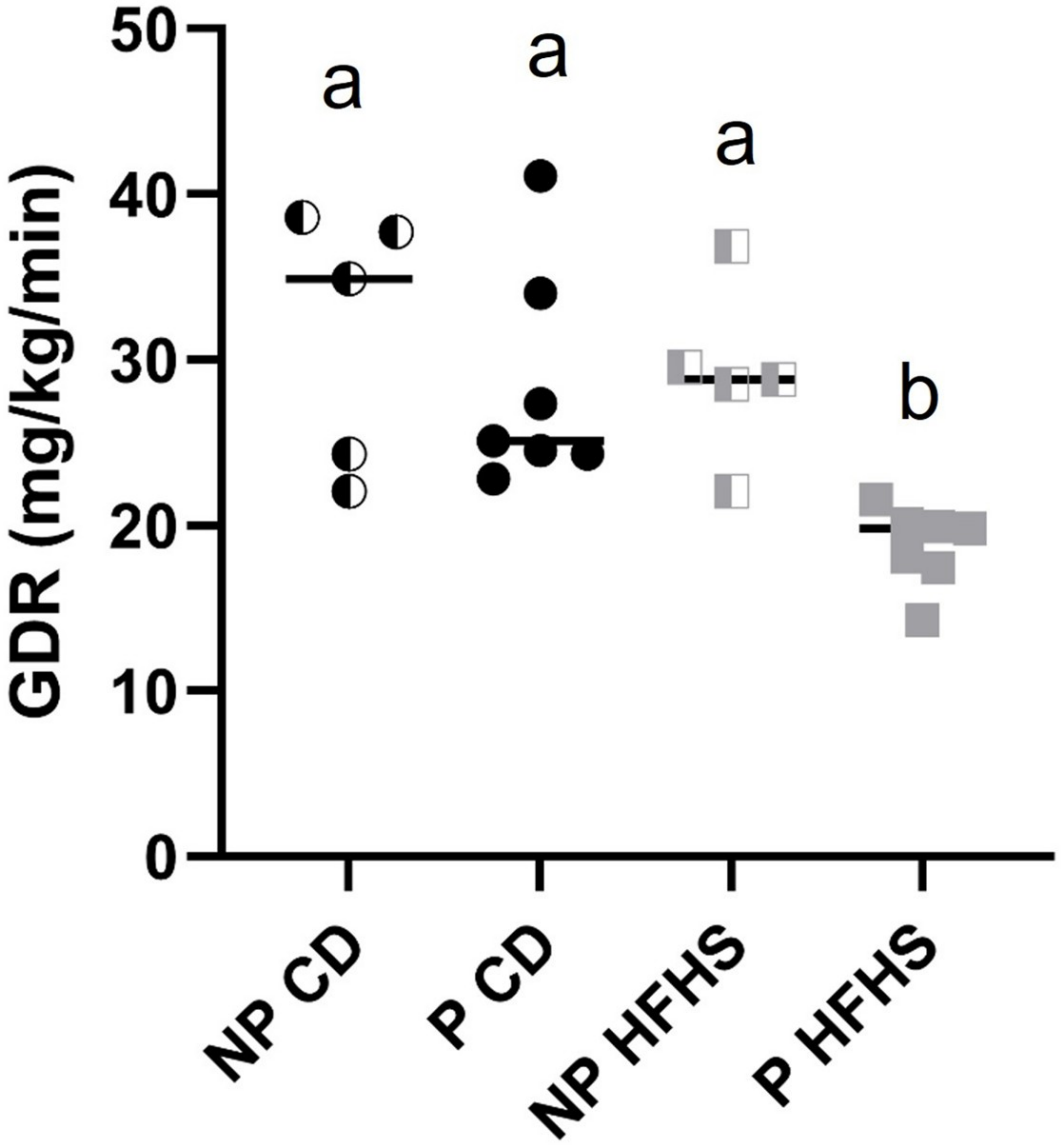
Glucose disposal rate (GDR) is significantly decreased in P HFHS dams. Glucose disposal was measured at time of hyperinsulinemic euglycemic clamp studies. GDR was significantly reduced in P HFHS dams compared to all other groups. N = 5 NP CD, 7 P CD, 5 NP HFHS and 7 P HFHS mice. Lines represent median values. Different letters indicate significant difference among groups (p<0.01).

By day 13.5 of pregnancy acute HFHS dams develop glucose intolerance [21], but insulin resistance has not been assessed in this model. Therefore, we performed hyperinsulinemic euglycemic clamp experiments, the gold standard for evaluating insulin resistance [23], at day 13.5 of pregnancy in P CD and P HFHS dams. Insulin resistance was also assessed in NP females on either CD or HFHS diet for the equivalent time to P CD and P HFHS dams in order to distinguish the effects of diet alone on insulin resistance versus the combined effect of diet and pregnancy (see Fig 3A for experimental design). Glucose infusion rate (GIR) at clamped condition (Fig 3B) and glucose infusion rate over time (Fig 3C) were significantly reduced (p<0.05) in P HFHS dams in compared to NP HFHS as well as to all other groups, indicating the development of an insulin resistance phenotype specific to P HFHS dams compared to all other groups. GIR over time was also significantly reduced (p<0.05) in P CD in compared to NP CD, consistent with a pregnancy-dependent development of insulin resistance. GIR also significantly decreased in NP HFHS compared to NP CD but was not different between P CD and NP HFHS dams (Fig 3C), suggesting a modest effect of the diet in the absence of pregnancy. Blood glucose levels during the time of the clamp procedure were not significantly different among groups (S1 Fig). There was a significant effect (p<0.02) of pregnancy in combination with diet on glucose disposal rate (GDR, Fig 4) with P HFHS dams having reduced GDR compared to all other groups. Taken together, these results indicate that a HFHS fed during pregnancy results in insulin resistance as indicated by reduced glucose infusion and glucose disposal rates and that this insulin resistance is specific to P HFHS dams.

### Glucose production is less suppressed in P and HFHS dams

**Fig 5:**
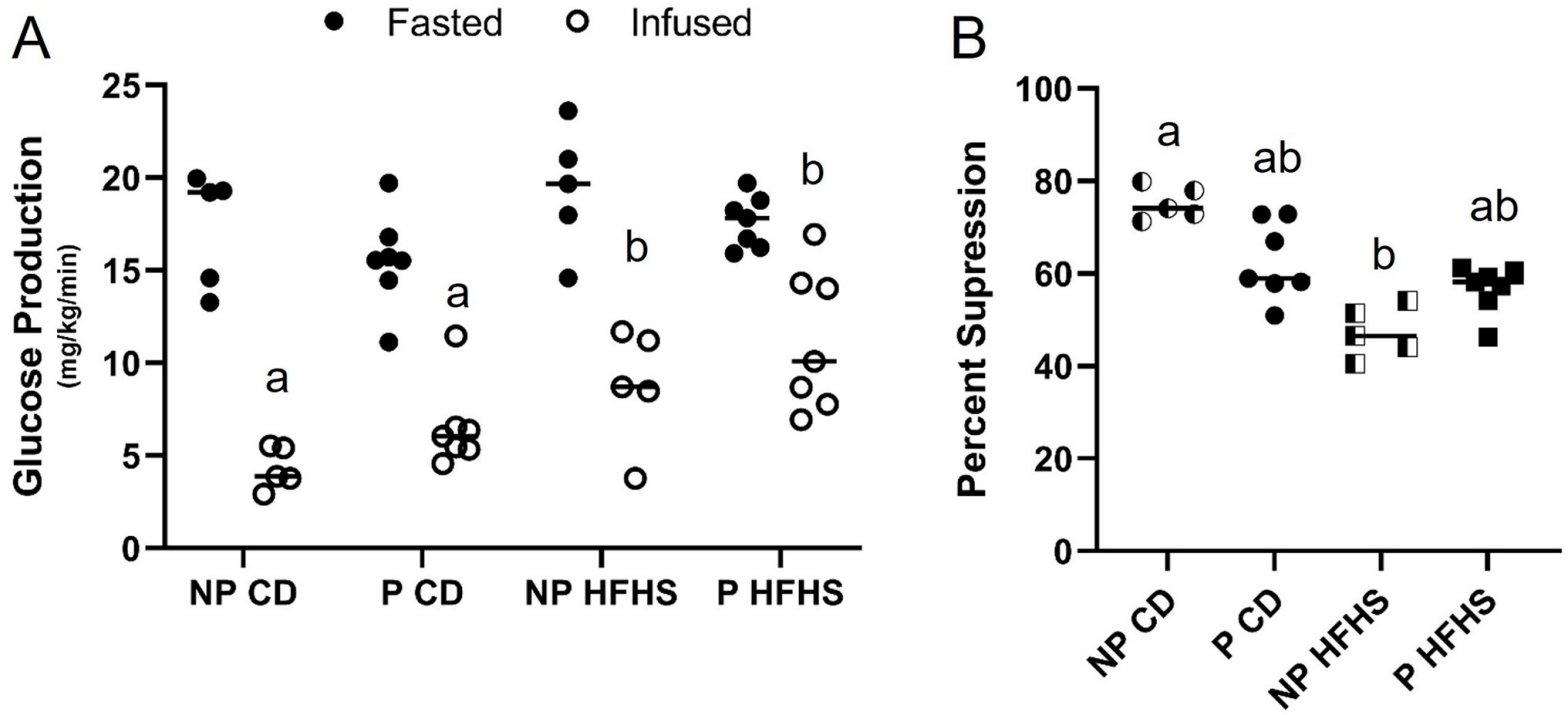
Glucose production is suppressed by HFHS diet. Glucose production was obtained during the fasted and infused state and then percent glucose suppression was calculated. (A) Glucose production in the basal and clamped state. (B) Percent suppression of glucose production from basal to clamped state. N = 5 NP CD, 7 P CD, 5 NP HFHS and 7 P HFHS mice. Lines represent median values. * represents p<0.01. Different letters indicate significant difference among groups (p<0.01).

Basal glucose production (after 4 hours fast) was not different among groups (Fig 5A). Under hyperinsulinemic clamp condition, all the dams showed significant suppression of hepatic glucose production (p<0.01) compared to basal (Fig 5A). The percent suppression in glucose production from basal to clamped state was significantly (p<0.007) decreased by diet alone as well as pregnancy status (p<0.02) however there was no significant interaction between pregnancy status and diet (Fig 5B). P HFHS dams had decreased glucose suppression compared to NP CD females (p<0.05), but not P CD females. Blood glucose levels at time of tissue collection, 45 minutes post 2-deoxyglucose injection, were not different among groups (S2 Fig). Taken together these results suggest that acute exposure to a HFHS diet right before and during pregnancy suppresses hepatic insulin sensitivity and may be one important factor for developing GDM.

### Acute HFHS feeding results in increased fetal and placental glucose uptake

**Fig 6:**
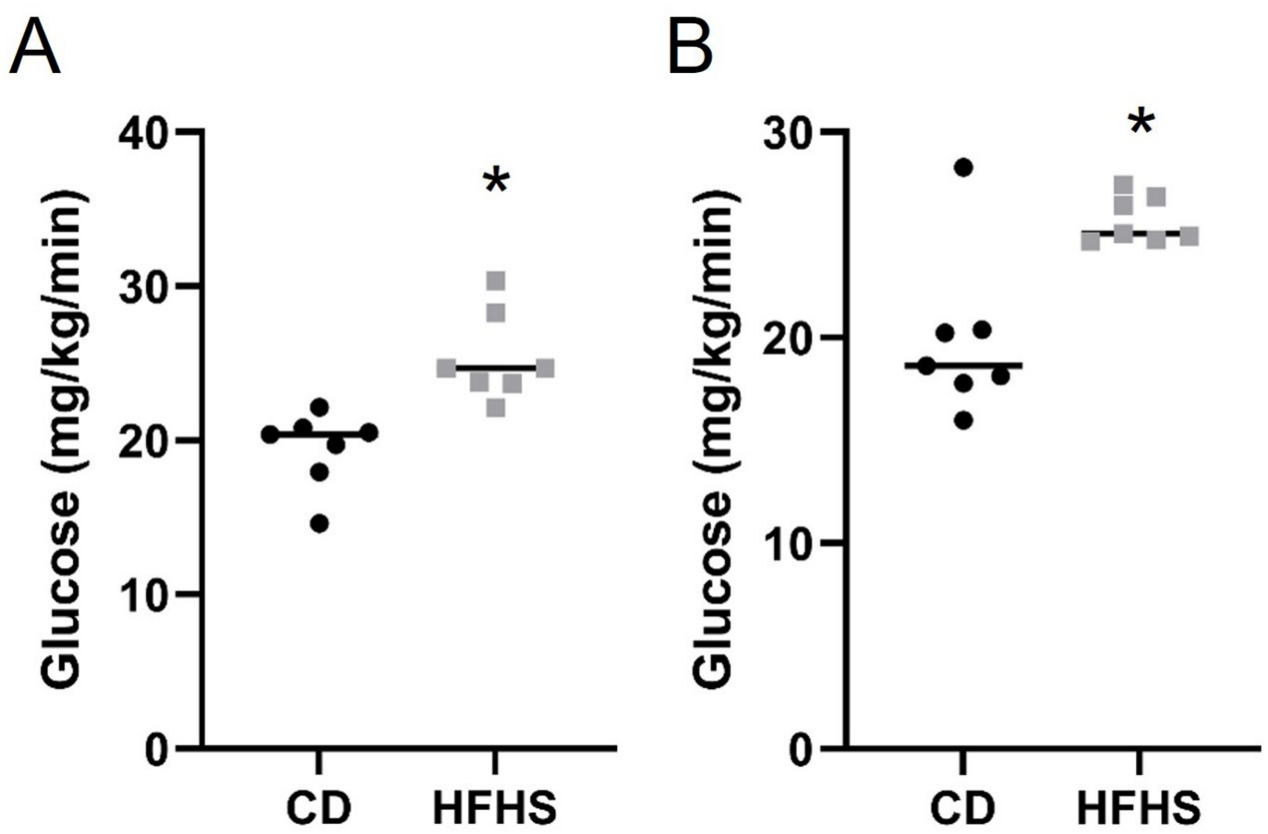
Fetal (A) and Placental (B) glucose uptake are increased in P HFHS dams. Lines represent median values. N = 7 dams per group. * represents p<0.01.

Following glucose clamp procedures, fetal and placental tissue were collected, and glucose uptake was assessed. Fetal (Fig 6A) and placental (Fig 6B) glucose uptake were significantly increased (p<0.05) in HFHS dams compared to CD dams. Thus, the increased maternal glucose concentrations and reduced uptake of glucose by the maternal system is accompanied by increased transfer of glucose to the placenta and fetus.

Previously we reported that there were no fetal or placental weight differences at day 13.5 or 17.5 of pregnancy [21], however when previous data was combined with data collected from experiments in this manuscript, we found that fetal weights were significantly increased (p = 0.014) at day 17.5, but not 13.5, of pregnancy in P HFHS compared to P CD fetuses (Table 1). No differences were observed in placental weights at either time point (Table 1). Increased fetal weights at day 17.5 maybe in part due to increased fetal and placental glucose uptake. Furthermore, this data corresponds with the known macrosomia observed in GDM fetuses.

**Table 1:** Combined Fetal and Placental Weights at day 13.5 and 17.5 of pregnancy.

	P CD	N	P HFHS	N	P Value
**Day 13.5**					
Maternal (g)	27.08±0.44	11	27.33±0.35	14	.779
Fetus (g)	0.180±0.009	11	0.193±0.011	14	.399
Placenta (g)	0.099±0.005	11	0.100±0.003	14	.738
Pup Number	8.18±0.44	11	8.57±0.39	14	.515
**Day 17.5**					
Maternal (g)	30.09±0.45	13	31.06±0.89	12	.429
Fetus (g)	0.863±0.013	13	0.935±0.024*	12	.014
Placenta (g)	0.111±0.004	13	0.107±0.003	12	.549
Pup Number	7.15±0.45	13	7.08±0.51	12	.918

Fetal and placenta weights were averaged per dam and n corresponds to the number of dams per group. ± standard error;

* indicates P<0.05

### Glucose stimulated insulin production is diminished in P HFHS dams by mid pregnancy

**Fig 7:**
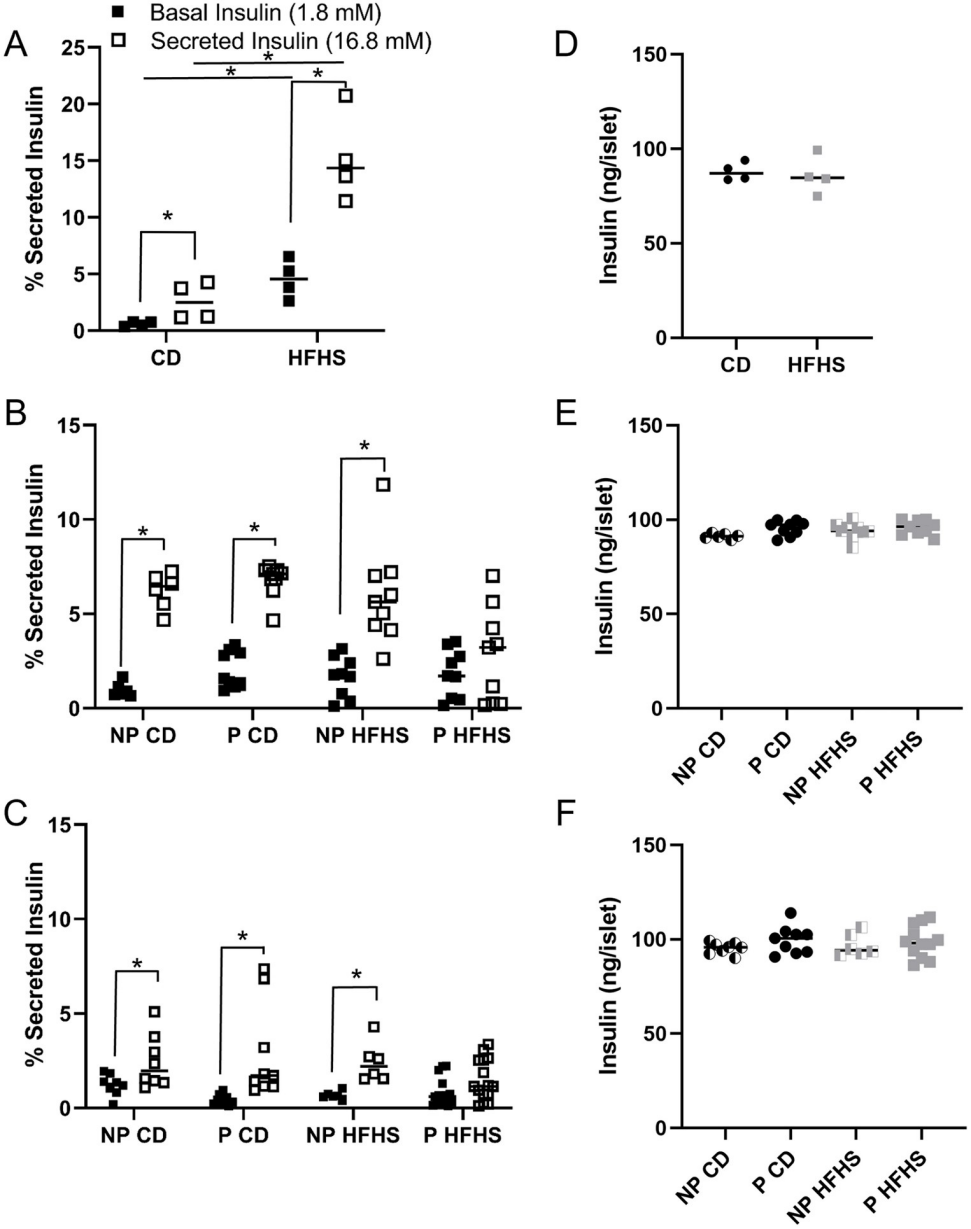
Beta cell function is diminished in P HFHS. Glucose stimulated insulin secretion (GSIS) is high at basal and blunted in response to glucose in P HFHS dams by mid pregnancy (Fig C). Insulin secretion in the basal and secreted state in Fig A-C and total insulin in Fig D-F at day 0 (Fig A and D), 13.5 (Fig B and E) and 17.5 (Fig C and F) of pregnancy. Day 0 n = 4 CD and 4 HFHS mice, day 13.5 n = 6 NP CD, 9 P CD, 9 NP HFHS mice, and 9 P HFHS, day 17.5 n = 8 NP CD, 9 P CD, 6 NP HFHS, and 15 P HFHS mice. Lines represent median values. * represents p<0.05.

We have previously shown that beta cell numbers and serum insulin levels are decreased in P HFHS dams at mid (day 13.5) and late (day 17.5) of pregnancy (Fig 2G) [21]. However, it is unknown if decreased insulin levels are due to decreased beta cell numbers alone or a combination of decreased beta cell response to glucose stimulation and decreased beta cell numbers. To address this, we performed glucose stimulated insulin secretion (GSIS) assays on isolated pancreatic islets at day 0, 13.5 and 17.5 of pregnancy to determine if insulin secretion was diminished in P HFHS dams. GSIS assays at day 0, CD-fed females had significantly increased insulin secretion in response to glucose (P<0.05) as expected (Fig 7A). Basal insulin secretion was high for female mice on HFHS diet (P<0.05) and it further increased (P<0.05) when stimulated with high glucose compared to CD females (Fig 7A). At day 13.5 and 17.5, GSIS was performed on NP CD, P CD, NP HFHS and P HFHS animals. At day 13.5, NP CD, P CD and NP HFHS islets showed increase in insulin secretion in response to glucose (Fig 7B). However, P HFHS diet showed blunted insulin secretion in response to glucose and had significantly lower secreted insulin compared to P CD dams, but not NP CD and NP HFHS females (Fig 7B). At day 17.5, NP CD, P CD and NP HFHS dams showed a significant (P<0.05) increase in insulin secretion in response to glucose but again P HFHS dams showed a blunted response (Fig 7C). Total insulin was not different among groups at day 0, 13.5 or 17.5 of pregnancy (Fig 7D–7F). Take together this data shows GSIS is not impaired in HFHS fed females on day of mating, however GSIS is decreased in P HFHS dams by mid pregnancy while total insulin is not affected at day 0, 13.5 or 17.5 of pregnancy. Taken together, P HFHS dams decreased serum insulin levels are due to both decreased beta cell numbers and inhibited GSIS indicating diminished beta cell function in P HFHS dams by mid pregnancy.

## Discussion

Previously we demonstrated that acute exposure to a HFHS diet 1 week before pregnancy and throughout gestation results in glucose intolerance, decreased beta cell numbers and serum insulin levels, as well as dyslipidemia during pregnancy [21, 22]. Here we evaluated the effects if acute HFHS feeding on insulin resistance and beta cell function. We showed that acute HFHS feeding results in insulin resistance in the form of reduced glucose disposal in response to insulin, accompanied by increased glucose transfer to the fetus which may contribute to increased fetal weights observed at d17.5 of pregnancy. We also showed that on day 0 of pregnancy insulin sensitivity, beta cell numbers and beta cell function are not impaired by acute HFHS diet feeding. Finally, we demonstrated that acute HFHS diet feeding impairs beta cell function by mid pregnancy and that these responses to HFHS feeding are specific to pregnancy. Together the data presented here, and our previously published work demonstrate that acute HFHS diet exposure results in GDM in mice. This non-obese GDM mouse model will be useful in determining the underlying mechanisms for the pathophysiology of GDM and could be used to test novel therapies for the treatment of GDM.

Women with GDM have suboptimal beta cell expansion in response to the natural state of insulin resistance during pregnancy [2, 9, 12]. Previously we demonstrated that the acute HFHS mouse model mimics this lack of beta cell expansion by mid pregnancy [21]. Here we show that this decrease in beta cell numbers is not present at day 0 of pregnancy (Fig 2). Together this data suggests that pregnancy specific hormones, such as those produced by the placenta, may be dysregulated in our GDM mouse model. Placental hormones are major regulators of pregnancy associated beta cell expansion, particularly prolactin (Prl) and placental lactogen (Pl), [34–36]. These hormones function through the prolactin receptor (Prlr), which is expressed by mouse beta cells [37]. Mice heterozygous for Prlr (Prlr^+/-^) have a reduction in beta cell expansion and impaired glucose tolerance during pregnancy [37, 38]. Future work is planned to determine the specific mechanisms regulating beta cell expansion in our GDM mouse model.

Insulin resistance is a hallmark of GDM, however as Catalano reviewed, differences exist in insulin resistance between lean and obese women with GDM [39]. Lean women have reduced insulin sensitivity (insulin resistance) pre- and early pregnancy, but not in late pregnancy, as well as a reduction in first phase insulin release [39]. In contrast, obese women have reduced insulin sensitivity throughout pregnancy, and no alteration to first phase insulin secretion [39]. Although obesity is an established risk factor for developing GDM, less than half of women with GDM are obese with a BMI >30, and 29.3% of women with GDM have a BMI of <25 [8]. Furthermore, Powe et al demonstrated in a large cohort of pregnant women, that there was heterogeneity of the physiological processes underlying GDM, with one third of GDM women having predominantly impaired insulin secretion defects without impaired insulin sensitivity, and one half of GDM women having predominantly insulin sensitivity defects with hyperinsulinemia. Together these human studies demonstrate the need to have animal model tools to understand the mechanisms affecting both insulin secretion defects and impaired insulin sensitivity during pregnancy. Here, we demonstrate our non-obese mouse model displays insulin resistance (Figs 3–5) and impaired insulin secretion defects (Fig 7) making it an ideal model for studying the pathophysiology of GDM.

The finding that placental and fetal glucose uptake in increased at day 13.5 of pregnancy in GDM dams (Fig 6) suggests one possible mechanism by which GDM alters the future health of offspring. Children born to mothers with GDM are at an increased risk for macrosomia at birth and developing obesity and type II diabetes later in life [2], and offspring of the HFHS fed mouse dams are likewise predisposed to greater adipose tissue deposition, as well as germ cell developmental defects [40–42]. Here we also show that like in humans, fetuses in our model have increased weights late in pregnancy (Table 1) indicating a macrosomic phenotype. Previously we have also shown that P HFHS dams have increased leptin and triglyceride levels as well as increased lipolysis, which can lead to increased free fatty acids which are also thought to impact fetal growth and lead to macrosomia [21, 22]. Additionally, studies have shown that controlling glucose levels in GDM patients does not eliminate macrosomia in offspring, increased free fatty acids also observed in women with GDM have been implicated in a likely pathway for increased fat mass in offspring born to women with GDM [43–45]. Nonetheless, the increased availability of glucose observed in this study may affect the development of energy regulating systems in the fetus, leading to the observed postnatal changes in adipose mass [40]. The data presented here along with our previously published work on dyslipidemia indicate our model maybe a useful tool in studying mechanisms of fetal macrosomia and the lasting health consequences associated with GDM.

Finally, we investigated the effects of acute HFHS diet 1 week before and during pregnancy on beta cell function, assessed by measuring total and glucose stimulated insulin secretion. We found that 1 week of HFHS feeding resulted in both increased basal and secreted insulin compared to control animals (Fig 7A). However, by mid pregnancy P HFHS dams had blunted glucose stimulated insulin secretion compared to all other groups and this finding continued through late pregnancy (Fig 7C and 7E). Previous reports have shown that in male mice long term HFHS feed for 6–12 weeks can increase basal insulin levels and decrease glucose stimulated insulin secretion, however the effects of 1 to 4 weeks of HFHS feeding were not reported [46]. Placental lactogens, working through the prolactin receptor are known to stimulate glucose stimulated insulin secretion [47]. Downstream of placental lactogens, serotonin working through the 5-HT3 receptor (Htr3), depolarizes pancreatic beta cells which enhances glucose stimulated insulin secretion during pregnancy [48]. Future work will examine the signaling pathways of placental lactogens to determine their role in the beta cell dysfunction observed in our mouse model of GDM.

Our acute HFHS mouse model of GDM has multiple advantages as it mimics many of the physiological characteristics of human GDM. As we have previously demonstrated HFHS dams display glucose intolerance specific to pregnancy, decreased serum insulin levels and beta cell numbers, dyslipidemia, and long-term maternal health complications [21, 22]. Here we demonstrate that HFHS dams also display insulin resistance specific to pregnancy (Fig 3) and beta cell dysfunction as demonstrated by decreased GSIS (Fig 7). However, limitations to this animal model, as with many animal models, exist. In women there are multiple risk factors for developing GDM including diet and lifestyle, pre-pregnancy BMI status and genetic background [1]. Nevertheless, less than half of women with GDM are obese, and an estimated 29.3% have a BMI <25 [8], highlighting the need for animal models which specifically focus on GDM with and without obesity. GDM in lean women is characterized by insulin resistance in the periconceptional period, and impaired insulin release in mid-late pregnancy. In contrast, obese GDM is associated with continuing insulin resistance and hyperinsulinemia [39]. Recently, Selen et al. showed that of women diagnosed with gestational glucose intolerance, 48% were insulin resistant, 27% were insulin deficient and 17% were both insulin resistant and insulin deficient [49]. These studies in women highlight the spectrum of GDM pathophysiology and the utility of our non-obese mouse model with both insulin resistance and insulin deficiency.

In conclusion, the data presented here definitively show that an acute exposure to HFHS diet 1 week before and during pregnancy results in insulin resistance and beta cell dysfunction by mid pregnancy, and that this effect is specific to pregnancy. Furthermore, our data shows that acute HFHS feeding also results in larger fetuses late in pregnancy and increased fetal and placental glucose uptake. These data further validate this animal model as a relevant model for GDM which can be used to study multiple aspects of the pathophysiology of GDM along with its long-term health consequences on mother and baby.

## Supporting information

S1 Fig Blood glucose concentrations during hyperglycemic euglycemic clamp procedures. Blood glucose levels were not different among groups during the duration of the clamp procedures. Lines represent median values. N = 5 NP CD, 7 P CD, 5 NP HFHS and 7 P HFHS.
(TIF)

S2 Fig Blood glucose concentrations at 45 min post 2-deoxyglucose administration. Blood glucose concentrations were not different among groups 45 minutes post 2-deoxyglucose administration (and time of tissue collection). Lines represent median values. N = 5 NP CD, 7 P CD, 5 NP HFHS and 7 P HFHS.
(TIF)

S1 File (XLSX)
