# Supplementary figures and images for: A novel model of gestational diabetes: Acute high fat high sugar diet results in insulin resistance and beta cell dysfunction during pregnancy in mice
Source: PLoS One. 2022 Dec 15;17(12):e0279041. doi: 10.1371/journal.pone.0279041 (PMC9754171; doi:10.1371/journal.pone.0279041)

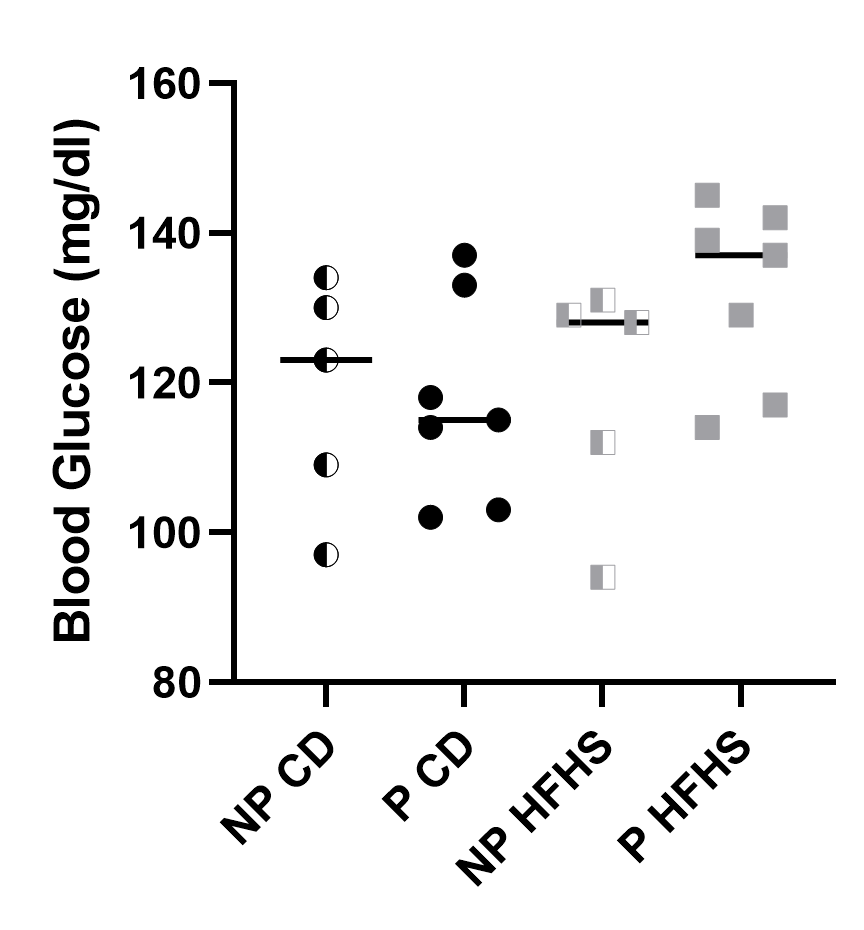

Supplement: S1 Fig — Blood glucose levels were not different among groups during the duration of the clamp procedures. Lines represent median values. N = 5 NP CD, 7 P CD, 5 NP HFHS and 7 P HFHS. (TIF) [file pone.0279041.s001.tif]

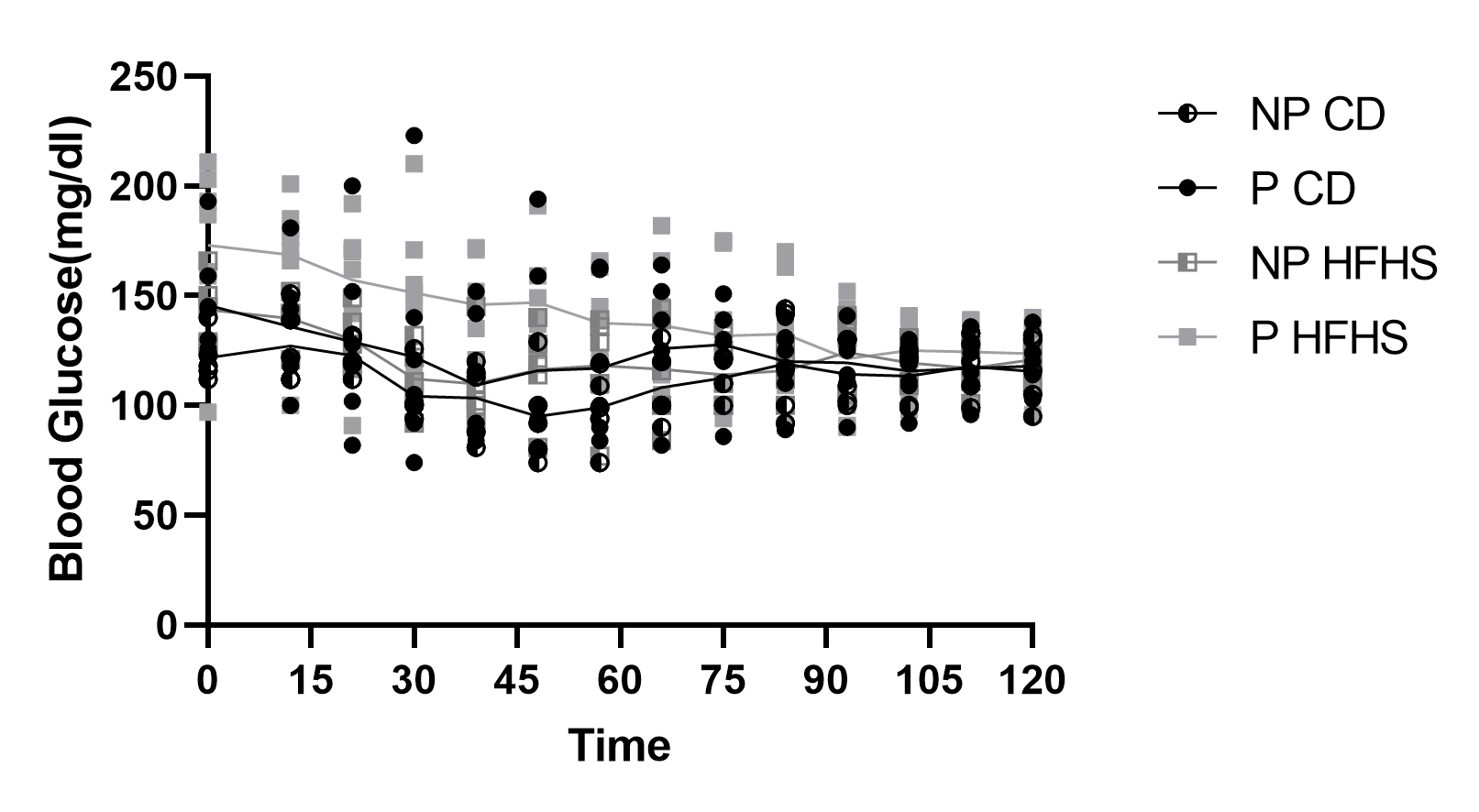

Supplement: S2 Fig — Blood glucose concentrations were not different among groups 45 minutes post 2-deoxyglucose administration (and time of tissue collection). Lines represent median values. N = 5 NP CD, 7 P CD, 5 NP HFHS and 7 P HFHS. (TIF) [file pone.0279041.s002.tif]
